# Primary umbilical endometriosis masquerading as strangulated umbilical hernia: a diagnostic pitfall

**DOI:** 10.1093/jscr/rjag395

**Published:** 2026-06-19

**Authors:** Syed Saad Ali Chishti, Adam Umair Ashraf Butt, Emeka Nzewi

**Affiliations:** Beaumont Hospital, Dublin D09V2N0, Ireland; Cavan General Hospital, Cavan H12Y7W1, Ireland; Cavan General Hospital, Cavan H12Y7W1, Ireland

**Keywords:** umbilical hernia, umbilical endometriosis, Villar's nodule, emergency surgery, cyclic umbilical pain

## Abstract

Primary umbilical endometriosis (Villar’s nodule) is a rare entity, representing 0.5%–1% of all endometriosis cases. We report a 37-year-old multiparous woman presenting with a 7-day history of umbilical pain, swelling, and constipation. Examination revealed a 2 × 2 cm hyperpigmented, firm, and tender umbilical nodule. The patient described cyclic umbilical pain over 5 months associated with an irregular menstrual cycle. Trans-abdominal ultrasound suggested an incarcerated umbilical hernia, leading to emergency surgery. Intraoperatively, a small hernial defect containing extraperitoneal fat and a separate subcutaneous nodule were identified without strangulation. Excisional biopsy of the nodule with primary hernia repair was performed. Histopathology demonstrated endometrial glands and stroma within fibroconnective tissue, confirming primary umbilical endometriosis. The postoperative course was uneventful, and the patient remained asymptomatic at 4-month follow-up. This case highlights diagnostic difficulty when umbilical endometriosis presents as a surgical emergency and emphasizes the importance of surgical excision with histopathological confirmation.

## Introduction

Endometriosis is a benign gynaecological condition that affects ~22% of females during their reproductive years [1]. It is defined by the presence of functional endometrial tissue and stroma in locations outside the uterine cavity. Endometriosis typically develops during the reproductive age because the endometrial tissue responds to hormones and undergoes cyclic changes during menstruation. After menopause, it usually resolves on its own [2].

Villar first described primary umbilical endometrioma in 1886, and it has since also been known as Villar’s Nodule [3]. Endometriosis is usually confined to the pelvic organs, causing chronic pelvic pain associated with dyspareunia and infertility.

We describe a case involving a 37-year-old woman who presented with symptoms of primary umbilical endometriosis that resembled a strangulated umbilical hernia.

## Case report

A 37-year-old female patient presented to the Acute Surgical Assessment Unit of our hospital with abdominal pain and tender swelling in the umbilical region for the past seven days. This was accompanied by constipation for the past 2 days.

The patient had a past medical history of irregular menstrual cycle for 2–3 years and polycystic ovarian disease. She had been experiencing cyclic, intermittent pain at her umbilicus for the past 5 months, associated with her irregular menstruation for which she took over the counter analgesics. One year ago, an magnetic resonance imaging (MRI) was done in a different hospital system following a routine ultrasound (USG), which showed a haemorrhagic cyst in the left ovary which was managed conservatively.

On examination, a nodular swelling measuring 2 × 2 cm was noted around the lower edge of the umbilicus. This swelling was hyperpigmented, firm, immobile, tender, irreducible, and tethered to the skin. Trans-abdominal USG scan revealed an umbilical hernia containing intra-abdominal fat, located immediately to the right of the umbilicus. This was reported as being relatively narrow necked, with the neck measuring 3 × 4 mm in diameter. A provisional diagnosis of incarcerated umbilical hernia was made, and the patient was scheduled for emergency hernia repair.

A transverse infraumbilical incision was made. Oedema within the subcutaneous tissue was noted with a small hernial defect containing extraperitoneal fat. There was a separate subcutaneous nodule. There were no features of strangulated hernia. Excisional biopsy was performed, along with simple umbilical defect repair, and the sample was sent for histopathological examination.

**Figure 1:**
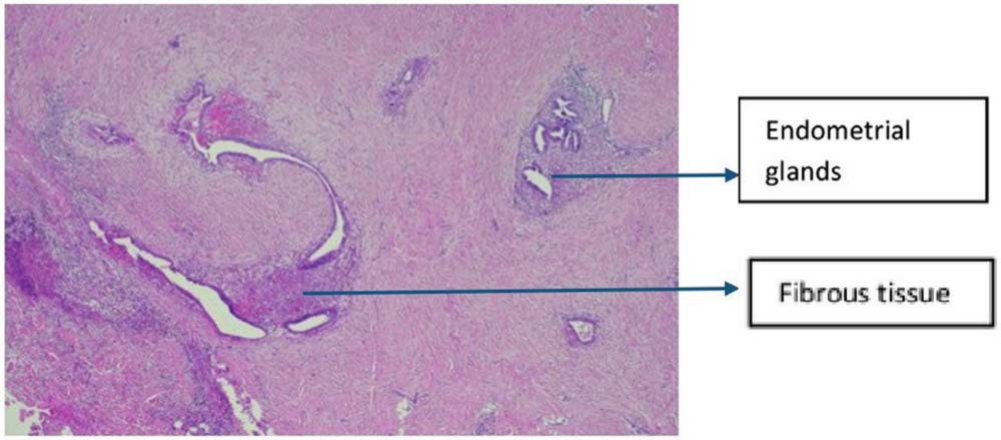
Histology of endometriotic nodule at 4× magnification.

**Figure 2:**
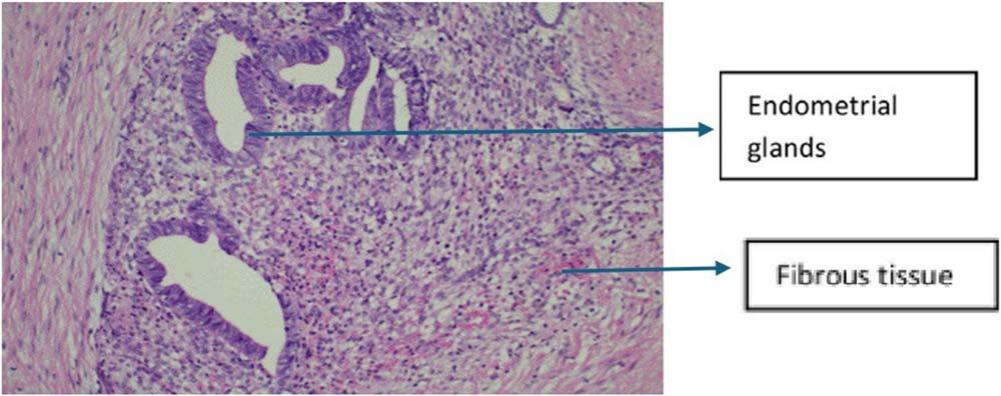
Histology of endometriotic nodule at 20× magnification.

Gross examination showed a firm ovoid nodule, 1.8 × 1.5 × 1.3 cm with a brown cut surface. Histopathology sections (Figs 1 and 2) showed endometrial glands and stroma embedded within fibroconnective tissue. The features are consistent with abdominal wall endometriosis.

She recovered well postoperatively and was deemed fit for discharge on postoperative day 2. She was reviewed in the clinic 4 months later.

## Discussion

Endometriosis is the occurrence of endometrial-like tissue in any extra-uterine cavity. It is frequently confined to the pelvic organs; however, it can be found in extra-pelvic organs such as gastrointestinal regions as well. The abdominal wall is the most common location amongst extra-pelvic endometriosis [4]. One of its variants, the umbilical endometrioma, also known as Villar’s nodule, is a rare presentation, typically found in 30%–40% of cases involving abdominal wall endometriosis and merely 0.5%–1% of all endometriosis cases [4, 5].

Primary spontaneous umbilical endometriosis within the umbilicus is very rare, usually occurring secondary to surgical scars and post-laparoscopy at the port site. However, in some rare instances, it might appear as a primary or spontaneous lesion as well [3]. Our case report describes a rarity in reported medical literature, which of a spontaneous umbilical endometriosis associated with an umbilical hernia.

Umbilical endometriosis is considered a diagnostic challenge for clinicians, particularly when associated with umbilical hernia. This is an exceptionally rare condition, as demonstrated in our patient’s case, which presented a diagnostic challenge due to its striking resemblance to a strangulated umbilical hernia. While an associated umbilical hernia is typically identifiable during physical examination, our case was particularly interesting because the patient presented with a triad of symptoms: umbilical swelling, localized pain, and constipation, raising initial suspicion of hernia incarceration.

Imaging modalities such as computed tomography (CT) scans, MRI, USG, and laparoscopy have long been employed to detect pelvic endometriosis [2].

In our case, the clinical presentation closely mimicked a strangulated hernia. Given the clinical presentation of acute umbilical pain with constipation and USG findings suggestive of incarcerated hernia, emergency surgical intervention was prioritized over further imaging such as CT scan. The definitive diagnosis was confirmed postoperatively through histopathological examination, highlighting the critical role of surgical exploration and tissue analysis in such diagnostically complex cases.

Endometriosis treatment varies depending on the location and severity of the disease. However, when a woman of reproductive age presents with a painful umbilical swelling, clinicians should include umbilical endometriosis in their differential diagnosis [3].

A systematic review revealed that 73.1% of patients diagnosed with umbilical endometriosis had no prior history of endometriosis and that the pain was the most common symptom that was present in 77.93% of the patients [5].

There is a 5.4% to 27% risk of recurrence in umbilical endometriosis after surgical excision [5]. However, excellent results have been achieved by extended surgical resection which appears to significantly reduce likelihood of recurrence, particularly with the primary closure associated with small hernial defect. A good takeaway lesson was that when a young female patient presents with a complaint of umbilical swelling associated with pain, umbilical endometriosis should be considered, and further imaging and evaluation should be done [5, 6].

Another treatment point is that if a nodule is noted in subcutaneous tissue, like in our case, the nodule should be excised with margins and primary repair be done if the defect is <2 cm. However, if there is a recurrent hernia or the defect is >2 cm, mesh repair should be considered [1].

## Conclusion

In women of reproductive age who present with a complaint of umbilical swelling, it is important to inquire about gynaecological history, particularly the presence or absence of the cyclic nature of symptoms and consider advanced imaging based on provisional diagnosis.
